# Kidney retrieval after sudden out of hospital refractory cardiac arrest: a cohort of uncontrolled non heart beating donors

**DOI:** 10.1186/cc8022

**Published:** 2009-08-28

**Authors:** Fabienne Fieux, Marie-Reine Losser, Eric Bourgeois, Francine Bonnet, Olivier Marie, François Gaudez, Imad Abboud, Jean-Luc Donay, France Roussin, François Mourey, Frédéric Adnet, Laurent Jacob

**Affiliations:** 1 Department of Anesthesia and Critical Care, Hôpital Saint-Louis, Assistance Publique-Hôpitaux de Paris, Université Paris-7 Diderot,1 Avenue Claude Vellefaux, 75010 Paris, France; 2 Department of Urology, Hôpital Saint-Louis, Assistance Publique-Hôpitaux de Paris, Université Paris-7 Diderot,1 Avenue Claude Vellefaux, 75010 Paris, France; 3 Department of Nephrology, Hôpital Saint-Louis, Assistance Publique-Hôpitaux de Paris, Université Paris-7 Diderot,1 Avenue Claude Vellefaux, 75010 Paris, France; 4 Department of Microbiology, Hôpital Saint-Louis, Assistance Publique-Hôpitaux de Paris, Université Paris-7 Diderot,1 Avenue Claude Vellefaux, 75010 Paris, France; 5 Organ Transplant Coordination Team, Hôpital Saint-Louis, Assistance Publique-Hôpitaux de Paris, Université Paris-7 Diderot,1 Avenue Claude Vellefaux, 75010 Paris, France; 6 Department Samu 93, Hôpital Avicenne, Assistance Publique-Hôpitaux de Paris, Université Paris-13, 125, rue de Stalingrad, 93000 Bobigny, France

## Abstract

**Introduction:**

To counter the shortage of kidney grafts in France, a non heart beating donor (NHBD) program has recently been implemented. The aim of this study was to describe this pilot program for kidney retrieval from "uncontrolled" NHBD meaning those for whom attempts of resuscitation after a witnessed out-of-hospital cardiac arrest (CA) have failed (Maastricht 1 and 2), in a centre previously trained for retrieval from brain dead donors.

**Methods:**

A prospective, monocentric, descriptive study concerning NHBD referred to our institution from February 2007 to June 2008. The protocol includes medical transport of refractory CA under mechanical ventilation and external cardiac massage, kidney protection by insertion of an intraaortic double-balloon catheter (DBC) with perfusion of a hypothermic solution, kidney retrieval and kidney preservation in a hypothermic pulsatile perfusion machine.

**Results:**

122 potential NHBD were referred to our institution after a mean resuscitation attempt of 35 minutes (20–95). Regarding the contraindications, 63 were finally accepted and 56 had the DBC inserted. Organ retrieval was performed in 27 patients (43%) and 31 kidneys out of the 54 procured (57%) have been transplanted. Kidney transplantation exclusion was related to family refusal (n = 15), past medical history, time constraints, viral serology, high vascular *ex vivo *resistance of the graft and macroscopic abnormalities. The 31 kidneys exhibited an expected high delayed graft function rate (92%). Despite these initial results transplanted kidney had good creatinine clearance at six months (66 ± 24 ml/min) with a 89% graft survival rate at six months.

**Conclusions:**

This study shows the feasibility and efficacy of an organ procurement program targeting NHBD allowing a 10% increase in the kidney transplantation rate over 17 months. With a six months follow-up period, the results of transplanted kidney function were excellent.

## Introduction

Given that the healthcare policies regarding end-stage kidney failure in western countries are for less restrictive criteria for entering the kidney transplant list and increasing indications to treat end-stage kidney failure, there is a worsening imbalance between needs and availability of kidneys from cadaver donors. Despite well-functioning organ retrieval programs from brain dead donors (BDD) and living-related donors, the waiting list has lengthened over the years [1].

Organ transplantation programs from non heart beating donors (NHBD) have been implemented in many western countries [2-5], as well as more recently in France [6]. In contrast to many other countries, this organ retrieval program exclusively concerns uncontrolled donors [7] after sudden out-of-hospital refractory cardiac arrest.

This prospective, single-centre, descriptive study reports the first data from a cohort of uncontrolled NHBD referred to our Hôpital Saint-Louis from February 2007 to June 2008.

## Materials and methods

A nationwide procedure for kidney retrieval from NHBD was organised by a committee of experts (prehospital emergency, intensive care and transplantation teams). NHBD are classified based on the Maastricht criteria [7] depending on whether cardiopulmonary function ceases spontaneously in the absence (Maastricht 1) or presence (Maastricht 2) of advanced life support or in a BDD (Maastricht 4), or after a medical decision to withdraw life-sustaining therapy from a hospitalised patient (Maastricht 3). This classification in fact opposes 'uncontrolled NHBD' which are patients in whom attempts of resuscitation after a sudden cardiac arrest have failed (Maastricht 1 and 2 categories) and 'controlled NHBD' (Maastricht 3). In France, the procedure excluded Maastricht 3 donors [6].

The procedure was established under the authority of the *Agence de la biomédecine *and was conducted in compliance with the Helsinki declaration. It was approved by the Ethics Committee of the Agency (22 June, 2004) and by the National Academy of Medicine [8]. The program for kidney retrieval from NHBD that was initiated in our institution in 2006 (Hôpital Saint-Louis, a tertiary teaching hospital, Assistance Publique – Hôpitaux de Paris, France) was in strict agreement with the national protocol enacted by the *Agence de la biomédecine*. In this protocol, next of kin approval for organ donation was obtained prior to any inclusion of the patient in the procedure of organ retrieval. Our observational study did not require any additional intervention and subsequently no further consent from next of kin was requested [9]. The *Agence de la biomédecine *undertook a national census of these donors in order to provide in parallel an independent longitudinal follow up.

### Patients and protocol of care

**Figure 1:**
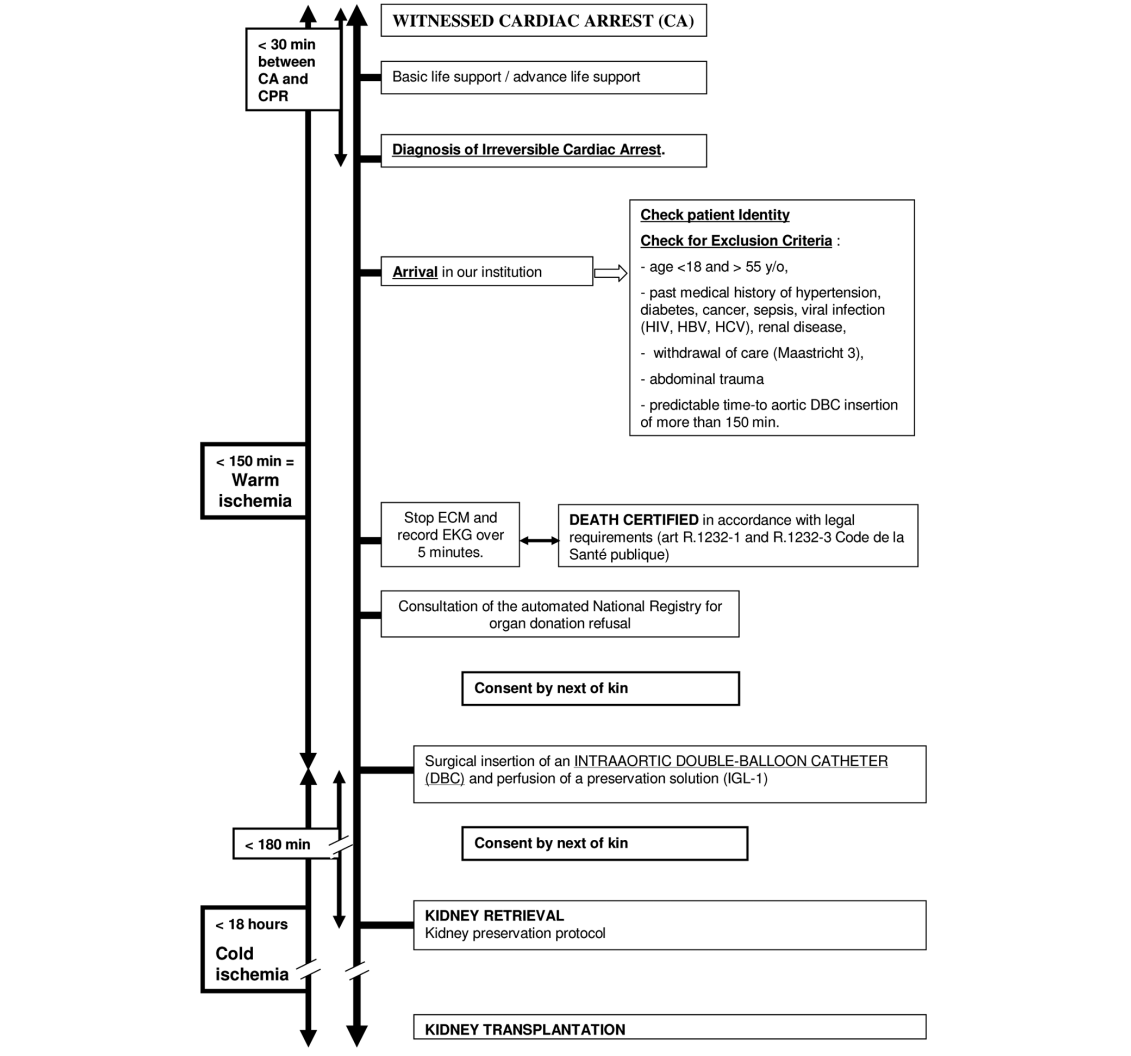
Protocol of care concerning non heart beating donors. Timings, exclusion criteria and protocol steps are described. The time between collapse and cardiopulmonary resuscitation (CPR) initiation had to be less than 30 minutes. The duration of CPR could not be less than 30 minutes. The time between collapse and intraaortic double balloon catheter (DBC) insertion had to be less than 150 minutes, defining warm ischaemia (WI). The time between DBC insertion and kidney retrieval had to be less than 180 minutes, while the kidney had to be transplanted within 18 hours after IGL-1 infusion initiation (cold ischaemia). HBV = hepatitis B virus; HCV = hepatitis C virus.

The protocol of care is fully described and timing limits are defined in Figure 1. Patients with out-of-hospital cardiac arrest were handled on site by the Fire Departments of Paris and suburbs for basic life support while the emergency medical services (such as *service d'aide medicale et d'urgence *(SAMU) from the departments 93, 95, 75, 92, 94 and 91) provided advanced life support [10,11]. These procedures were in accordance with the standard guidelines for cardiovascular pulmonary resuscitation (CPR) [12,13]. These cardiac arrests had to be witnessed to ascertain the time of collapse.

Upon contact with our institution, these patients were screened for eligibility by the coordination team according to demographic data and past medical history. Exclusion criteria are described in Figure 1. When the patients met the inclusion criteria, they were referred to our institution under mechanical ventilation and continuous external cardiac massage (ECM) machine (Autopulse, Zoll^®^, Chelmsford, MA, USA). Upon arrival, body temperature and end-tidal carbon dioxide were recorded. ECM was discontinued and echocardiogram was recorded over five minutes to check the absence of any spontaneous cardiac or haemodynamic activity. Death was certified in accordance with legal requirements [9] stating that the patient was unresponsive to nociceptive stimuli, showed no spontaneous motor activity, no respiratory effort, with an absence of brainstem reflexes. The automated National Registry for organ donation refusal was consulted.

Standard blood work was then performed as a conventional prerequisite for donation: type and screen, human leukocyte antigen typing and toxicology tests. Viral serologies were sent to the reference laboratory. In addition, blood cultures were performed in most patients and were analysed in the Microbiology Department. The blood culture results were compared with the occurrence of infections in recipients within the initial 15 postoperative days.

The possible aetiologies for cardiac arrest were investigated when possible (medical history, last symptoms, post mortem clinical examination, radiological or biological examination, autopsy).

### Kidney protection protocol

An intraaortic double-balloon catheter (DBC) and a venous vent were surgically inserted via an incision in the right side of the groin. After injection of 1.5 M U streptokinase, the arterial inlet was perfused with a fourth generation heparinised (5000 U/l) preservation solution (IGL-1^®^, Institut Georges Lopez, Saint-Didier-au-Mont-d'Or, France) at a rate of 20 litres within 180 minutes. After kidney retrieval, preservation protocol consisted in hypothermic (1 to 4°C) pulsatile perfusion over eight hours in KPS-1^® ^(Lifeport^®^, Organ Recovery System, Des Plaines, IL, USA). The organ preservation solution used in this device was provided by the manufacturer (UW solution, KPS-1^®^). The organ viability was assessed by measuring the *ex vivo *intrarenal vascular resistance [14,15]. Intrarenal vascular resistance had to be lower than 0.28 mmHg/mL/min. Kidneys with high initial resistance were transplanted if it normalised after one hour of pulsatile perfusion. A graft biopsy was performed, but the results were not available before the transplantation.

### Kidney transplantation criteria and protocol

Inclusion criteria for organ recipients were: age less than 60 years, no immunisation and signed informed consent (especially for the risk of delayed kidney function). A different waiting list had been opened for patients willing to join this NHBD program while remaining on the standard BDD list. Postoperative care and follow-up was standardised by the Nephrology Transplant Unit. Prophylactic antibiotic therapy with amoxicillin/clavulanic acid was administered for five days after transplantation and a routine check for infections was performed as per protocol. Immunosuppressive therapy used rabbit anti-human thymocyte globulin (thymoglobulins) and steroids for induction, mycophenolate mofetil and cyclosporine for maintenance. Delayed graft function was defined as the need for dialysis during the first week after transplantation with subsequent recovery of renal function. Data were expressed as mean ± standard deviation or as median (range).

## Results

### Cohort description

**Figure 2:**
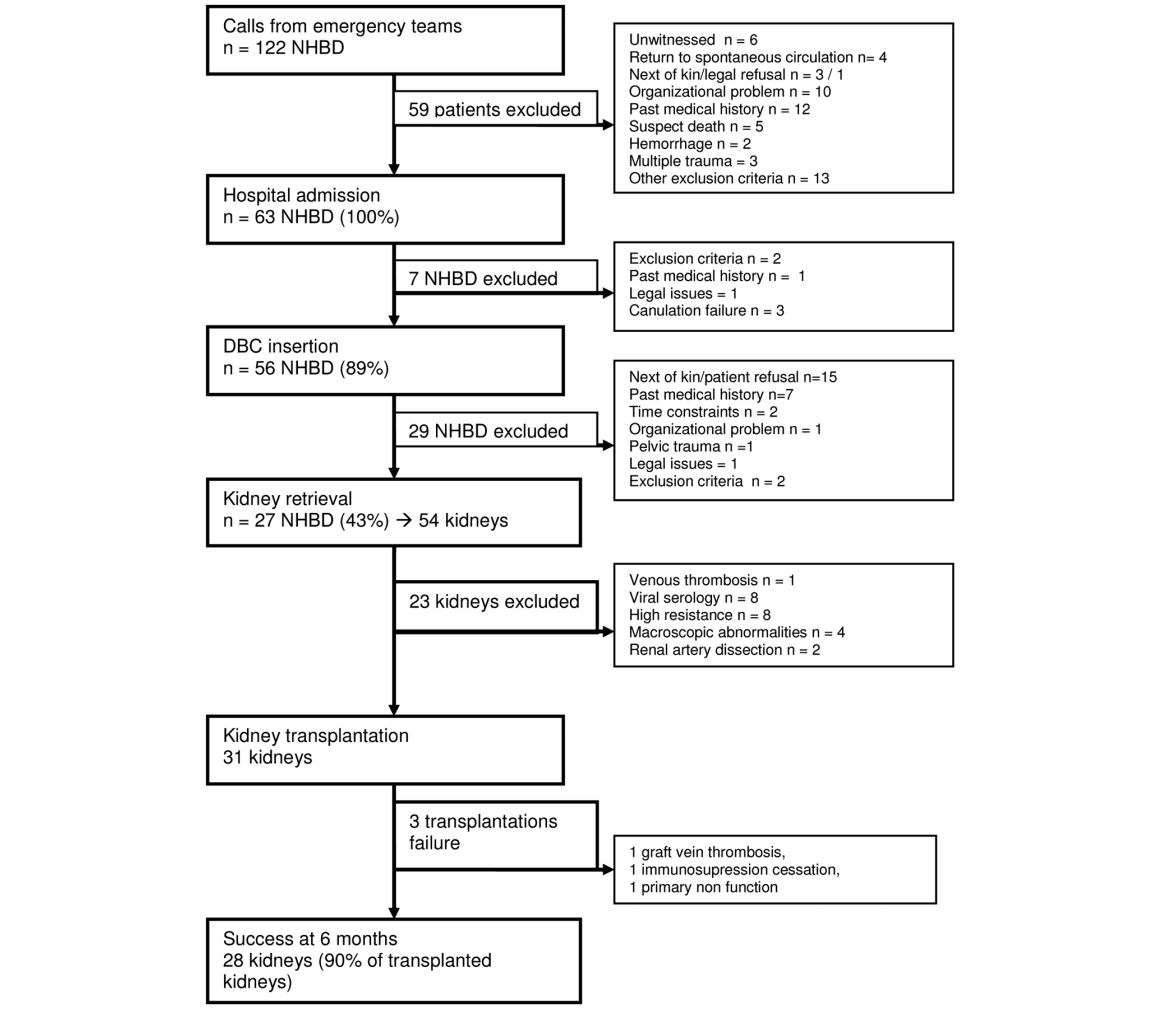
**Study profile of non heart beating donors**. DBC = double balloon catheter; NHBD = non heart beating donor.

From 1 February 2007 to 30 June 2008, 122 refractory cardiac arrests were screened in our institution. The demographic data of these potential donors showed mostly men (80%), with a mean age of 41.6 ± 11.6 years. Cardiac arrest occurred either at home (52%), outdoors (30%) or at work (16%). Among these, 59 (48.4%) did not meet inclusion criteria as shown in Figure 2. The main organisational problems were an overbooked intensive care unit (ICU) or surgeon unavailability (n = 8). Finally, 63 eligible NHBD (52%) were accepted for organ retrieval. Their main demographic and clinical characteristics are summarised in Table 1.

**Table 1:** Demographic, clinical and resuscitation characteristics of non heart beating donors admitted to the authors' institution (n = 63)

Male sex – n (%)	54 (86%)
Age – years	41 ± 10
Location of cardiac arrest – %	
Home	52.4%
Outdoors	30.2%
Workplace	15.8%
Psychiatric hospital	1.6%
Maastricht 1/2 – n (%)	54 (86%)/9(14%)
CPR performed by bystanders (%)	39%
Automated external defibrillation by emergency medical technicians	37%
Temporary return to spontaneous circulation during advanced life support – n (%)	5 (8%)
Duration of spontaneous circulation – minute (± SD)	11 ± 7
End-Tidal carbon dioxide at admission (n = 31) – mmHg	13 ± 12
Temperature at admission – °C	33 ± 2
Interval between phone SAMU call to ICU admission – minutes	48 (13–120)
Interval (minutes median (min-max)) from collapse	
To phone call from bystanders to the advanced life support	0 (0–28)
To external cardiac massage	5 (0–30)
To CPR for basic life support	10 (1–40)
To CPR for advanced life support	18 (0–56)
To discontinuation of unsuccessful CPR	35 (20–95)
To departure from the site	61 (35–130)
To arrival at our hospital	105 (65–163)
To aortic catheter placement	144 (105–185)
Cold ischaemia (DBC insertion to transplantation)-hours median (min-max)	12 h 52 (8 h 30–18 h 00)
Interval from aortic catheter placement to retrieval – minute median (min-max)	175 (110–225)

Data expressed as mean ± standard deviation (SD) unless stated otherwise.

CPR = cardiopulmonary resuscitation; DBC = double balloon catheter; ICU = intensive care unit.

### NHBD procedure

Thirty seven (59%) cardiac arrests occurred during the day time (8 am to 8 pm) and 26 during night duty (41%). Pre-hospital resuscitation is described in Table 1. External cardiac massage was performed within 5 (0 to 30) minutes, while automated external defibrillator was activated in 37% of the cases. Once the advanced cardiac life support team was on site, 17 (27%) patients presented with ventricular fibrillation. Five patients recovered a transient spontaneous cardiac activity for a mean duration of 11 ± 7 minutes. The mean interval to arrival at our institution after acceptance of NHBD was 53 ± 23 minutes. Among these 63 NHBD, aortic DBC was inserted in 56 NHBD (Figure 2). However, on retrospective analysis, time limit for DBC insertion exceeded the protocol requirement in 12 donors (21%) for a mean interval of 12 ± 11 minutes. Among these latter donors, 12 kidneys were retrieved and 6 were finally transplanted. Between aortic catheter insertion and kidney retrieval, 175 minutes (110 to 225) elapsed (Table 1). Thus, the interval exceeded 180 minutes in 6 patients (22%) for a mean period of 23 ± 19 minutes. Among those 12 kidneys, 5 were not transplanted due to positive HIV serology or high intra-renal *ex vivo *resistance.

### Cause of cardiac arrest

The probable or confirmed aetiologies of cardiac arrest are listed in Table 2. The aetiology was obvious for traumatic cases, some myocardial infarctions, aortic dissection during organ retrieval and when the post mortem medical examination could be performed. Thirteen autopsies were carried out. Seven were ordained by the legal authorities (access to the results was subsequently denied) and six medical autopsies were accepted by the surrogate decision makers. In four cases, autopsy provided diagnosis: two myocardial infarctions, one gastrointestinal haemorrhage secondary to a gastric ulcer and one mitral prolapse possibly responsible for sudden death. For the two remaining patients, the post mortem examination was negative.

**Table 2:** Death aetiologies of sudden cardiac arrest in 63 non heart beating donors

Cause	Confirmed	Probable
Trauma	13 (20.6%)	
Cardiac cause	6 (9.5%)	14 (22.2%)
Stroke	4 (6.3%)	2 (3.2%)
Aortic dissection	2 (3.2%)	1 (1.6%)
Hanging	2 (3.2%)	
Pancreatitis		1 (1.6%)
Hyperkalaemia		1 (1.6%)
Stab wound	1 (1.6%)	
Gastrointestinal bleeding	1 (1.6%)	
Meningitis	1 (1.6%)	
False passage	1 (1.6%)	
Unknown	13 (20.6%)	

Unknown cause was defined when clinical examination or biological data were negative and in the absence of prodromes, medical history or evidence from relatives.

Blood alcohol was positive in 11 NHBD, with 6 patients under 1 g/l and 5 with a higher level ranging from 1.24 to 3.47 g/l. Four eligible donors had positive viral serology (rapid technique) contraindicating organ transplantation at first analysis (HIV, human lymphocytes T virus (HTLV) 1, hepatitis C virus (HCV)). Only one HIV infection and one HCV infection were subsequently confirmed.

Blood cultures were performed in 44 NHBD, of which 30 were positive (68%). The origin of the isolated bacteria was from the gut in 16% cases (Gram-negative bacilli, anaerobes), the ears, nose or throat for 23% (Gram-positive streptococci and anaerobes) and skin for 61%. To differentiate a significant bacteraemia from a contamination, the following criteria were proposed: type of bacteria, aerobes or anaerobes and growth rate. Nineteen blood cultures were thus found to be positive, nine were contaminations and two were indeterminate. All blood cultures with bacteria originating either from the ears, nose, throat or gut were considered as clinically relevant. None of these bacteria was held responsible for infection in the recipients.

### Organ donation refusal

The family was present on site in 51% of cases. Death was declared on site in only 15 cases (24%) while the possibility of organ donation was proposed 13 times (21%). In all the other cases, this organ donation program was explained to the next of kin at our hospital. Among the 49 surrogate decision makers consulted for consent, 15 (31%) denied permission for organ donation: 3 transmitted the dead person advanced directives, while 12 refused it in the absence of or contrary to the donor's directives. Finally, 14 families (25%) were not consulted because of a contraindication to organ donation, a delay exceeding limits or failure to catheterise. Requests for permission of donation through the district attorney office in 25 NHBD (violent death) resulted in only 2 refusals. It was noteworthy that no refusal was recorded in the National Registry.

### Kidney retrieval and transplantation

Twenty seven eligible NHBD (43%) were finally retrieved (Figure 2). Among these 54 retrieved kidneys, 31 were transplanted and 23 kidneys were rejected mainly due to poor macroscopic appearance (4), positive HIV, HCV or HTLV serologies (8), venous thrombosis (1) or arterial dissection (2). Three out of the 4 rejected kidneys on account of poor macroscopic appearance had their 'twin' kidney transplanted with good results. In addition, eight kidneys were discarded because intra-renal vascular resistance was abnormally elevated during pulsatile perfusion.

**Figure 3:**
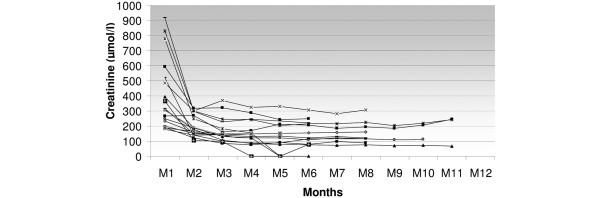
Serum creatinine individual evolution in the NHBD kidney recipients transplanted in the authors' institution (n = 21). Steady state creatinine level was obtained on average three months after transplantation. NHBD = non heart beating donor.

Among the 31 kidney grafts, 24 were transplanted in our institution and could enter our follow up. There was a rate of delayed graft function of 92%. The mean duration was 22 ± 9 days. Among these transplantations, three major complications led to graft loss: one untimely cessation of immunosuppressive therapy by the patient leading to acute rejection, one renal venous thrombosis with early graft removal, and one primary non function which may be related to longer warm ischaemia duration (185 minutes). The serum creatinine evolution is shown in Figure 3 for the remaining 21 patients. At three months, creatinine level was 162 ± 69 μmol/l and 152 ± 65 μmol/l at six months. Creatinine clearance at one month was 28 ± 14 ml/min, and 58 ± 21 and 66 ± 24 ml/min at three and six months after transplantation, respectively (n = 22). Graft survival rate was 89% at three and six months.

Limited information was available through the *Agence de la biomédecine *for six out of the seven recipients transplanted elsewhere. For a follow-up period ranging from 6 to 12 months, graft survival rate was 100% and mean serum creatinine level was 135 ± 53 μmol/l.

## Discussion

These data from uncontrolled NHBD showed that such a program was feasible in France and profitable in terms of successful organ transplantation. Indeed, even though only half of the out-of-hospital cardiac arrests that were proposed could enter this program and only one-quarter had their kidneys actually retrieved, this program provided at least 27 successful renal transplants, including 21 carried out at our institution, within 17 months.

Renal transplantation remains the treatment of choice for patients with end-stage renal failure [16]. In 2007 in France, 2911 kidney grafts were provided by BDD for 90.6%, by living donors for 8% and from NHBD for 1.4%. In 2007, 128 patients died on the waiting list for kidney transplantation. To counter the shortage of grafts, an alternative source was organ harvesting from NHBD. This procedure, previously described in Europe, Japan and the USA [2,3,5,17], concerned mainly Maastricht 3 category NHBD. If harvesting controlled donors (withdrawal of care) provokes ethical controversies [18-20], 'uncontrolled donors' triggers many organisational problems.

In our institution, the initiation of this program proved satisfactory in many ways. On account of the very strong implication of the prehospital emergency services, an important cohort of potential NHBD was rapidly recruited. Hypothermia and poisoning were excluded because they had to receive an extracorporeal life support in accordance with the standard guidelines for CPR [12,13]. The admission rate was high, and during this first 17 months of activity we included more NHBD than expected when compared with other European centres trained in this procedure [3,21,22]. Few countries perform organ harvesting exclusively from 'uncontrolled donors' in Maastricht category 1 and 2. In our institution, the on-duty critical care and surgical teams were in charge of this activity. However, only 8% of potential donors were refused because of organisational problems. During the study period, 31 kidney grafts were obtained from NHBD, 64 from BDD and 23 from living donors. Between 2006 and 2007, the transplantation rate increased by 10%. This increase was not as important as expected from the literature [19,23] because at the same time the incidence of BDD decreased for independent reasons.

The interview of potential organ donors' families is a legal requirement in France [6]. The National Registry did not yield any previous refusal, although this tool is only marginally used. We underwent a 32% rate of refusal and 15 potential donors were lost. This rate is in accordance with the national rate of refusal for BDD during 2007 (28%) but much higher than in Spain (between 7% [22] and 9.8% [24]). The reasons for refusal were primarily related to religious aspects, the wish to maintain an intact body or socio-cultural barriers in minority groups, as previously described [25]. When the family was present during resuscitation attempts (51%), the acceptance of death was easier. However, it was very difficult for the emergency team to discuss organ donation immediately after resuscitation as this could lead to confusion in the mind of the family.

Apart from family refusal, the transplantation rate was low: only 57.4% of the retrieved kidneys were transplanted, whereas in Spain the transplantation rate was more than 95% [3,22,26]. There are some differences in their procedure: in Spain they use partial cardiopulmonary bypass machines with external oxygenation and hypothermia or normothermia [22], whereas we used regional cooling with the DBC. Our grafts were preserved *ex vivo *on a pulsatile perfusion machine. The protocol took into account elevated vascular resistance, which has been a significant source of kidney exclusion in our study (22% retrieved kidneys). Sanchez-Fructuoso and colleagues [3] noticed that during their first 10 years of activity, they transplanted only 63% of their retrieved grafts of Maastricht type 1 and 2 donors. They used similar exclusion criteria except for high vascular resistance. In the future, the slope of the decreasing intrarenal resistance might also be considered for discarding organs.

It seems that all the teams whose procedure was similar to ours (30 minutes maximum of no-flow duration, use of DBC) had a significant number of potential grafts being discarded. The discard rate for uncontrolled donors in England was estimated to range between 50 and 65% [27]. Our final transplantation rate was 25% (63 potential NHBD/31 renal grafts).

The most critical issue in NHBD is the damage caused by prolonged warm ischaemia occurring between cardiac arrest and organ cooling. It results in delayed graft function or even in cortical necrosis leading to primary non function. In uncontrolled NHBD, warm ischaemia time may be difficult to assess [28]. The timings exceeded the limits fixed by the protocol in some donors: the duration between cardiac arrest and initiation of CPR exceeded 30 minutes in two donors (34 and 40 minutes). In some donors, the timing fixed by the protocol could not be strictly observed for several reasons. The causes were a long delay for the donor to be transferred to our hospital or a longer than expected procedure for intraaortic DBC insertion or for kidney retrieval. Interestingly, two discarded kidneys underwent histological examination at 158 minutes (rather than 150 minutes) of warm ischaemia and 225 minutes of time-to-retrieval (rather than 180 minutes). Both showed well-preserved renal parenchyma with only moderate tubular necrosis.

Ischaemia occurring during kidney procurement is shorter in living donors and longer in cadaverous donors and NHBD, but has minimal influence on long-term graft survival [27]. Our primary non function rate was 3.2%, similar to that found by other teams [3,4,27], including cohorts of 'controlled donors' [2]. The delayed graft function rate for NHBD transplants is higher than in heart beating donor (HBD) kidneys, and is more frequent in uncontrolled donors [21] than in controlled donors as illustrated by a greater incidence of acute tubular necrosis [29]. Patient survival and long-term graft function have been demonstrated to be equivalent in HBD and NHBD [2]. There is no difference for one year allograft survival and renal function is similar even after six years [29,30]. Thus, the high delayed graft function rate we observed (92%) was in accordance with the literature concerning uncontrolled NHBD [29,31]. Creatinine plasma levels were equivalent to those found by other teams in uncontrolled donors [3,31].

This procedure raised ethical controversies in France [32]. First, the question emerged about a conflict of interest between patient care and potential organ procurement. In this cohort, resuscitation duration was always longer than recommended. Secondly, to avoid any potential conflict of interest, there was a strict separation of roles between the care providers. The emergency physician in the SAMU ambulance independently considered the cardiac arrest to be irreversible and when to interrupt resuscitation manoeuvres. The intensivists were responsible for declaring death, approaching families while urologists and nephrologists dealt with recipient selection, subsequent organ harvesting and transplantation. The third point was that the legislation allowed *in situ *organ preservation by the introduction of a cooling device before family information [6] as in other countries. The rationale was to shorten warm ischaemia and to offer more opportunities to contact families for organ donation.

Recently, some teams argued for extending indications of extracorporeal circulatory assistance for out-of-hospital refractory cardiac arrest, similarly to hypothermic or poisoned patients [33] or some specific intrahospital cardiac arrests [34-36]. Inclusion criteria in this procedure needs to be defined and investigated because its efficacy remains uncertain for patients with out-of-hospital cardiac arrests [35].

## Conclusions

These data showed convincing results concerning kidney transplantation from NHBD. Strict adherence to the inclusion and exclusion criteria guarantees the long-term graft function. Although the rate of delayed graft function was almost 100%, results at three and six months were satisfactory and similar to those obtained by other teams involved in similar programs. NHBD programs on uncontrolled donors are challenging for transplant coordination teams. The procedure is a coordinated effort with participation of out-of-hospital emergency services and hospital staff. There is, however, a need for a better acceptance of organ donation by the population, which could be obtained by sustained nationwide information campaigns. This would also allow the emergency teams to approach the family on site, screening for potential consent.

## Key messages

• Patients dying from sudden out-of-hospital refractory cardiac arrests may be eligible to enter a highly standardised protocol of uncontrolled NHBD.

• This procedure elicited very different ethical issues compared with controlled Maastricht 3 donors (withdrawal of life sustaining therapy).

• Specific time and legal constraints of this emergency procedure implied a highly coordinated multidisciplinary teamwork in order to preserve organ function.

• Hypothermic pulsatile perfusion allowed prolonging *ex vivo *kidney resuscitation.

• Organ retrieval from uncontrolled NHBD may prove a valuable source of organs and is part of the answer to counter organ shortage, especially for the kidney.

## Abbreviations

BDD: brain dead donors; CPR: cardiovascular pulmonary resuscitation; DBC: double balloon catheter; ECM: external cardiac massage; HBD: heart beating donors; HCV: hepatitis C virus; HTLV1: human lymphocytes T virus; ICU: intensive care unit; NHBD: non heart beating donors; SAMU: *service d'aide medicale et d'urgence*.

## Competing interests

The authors declare that they have no competing interests.

## Authors' contributions

FF contributed to the implementation of this new procedure, was involved in data collection and analysis, and drafted the manuscript. MRL contributed to the design of the study, was involved in data analysis, and drafted and revised critically the manuscript. EB, FB and OM contributed to the implementation of this new procedure and were involved in data collection of the NHBDs. FG participated in the implementation of this new procedure and was involved in kidney retrieval and transplantation. IA was involved in the care and data collection of graft recipients and helped to draft the manuscript. JLD was involved in the microbiological procedures and data collection. FR and FM contributed to the implementation of this new procedure and to data collection, and were heavily involved in the family interviews. FA participated to the implementation of this new procedure and actively participated in patient inclusions. LJ contributed to the implementation of the new procedure and study design, and drafted and revised the manuscript.
